# Evaluation of the Protective Effect of Olive Leaf Extract on Cisplatin-Induced Testicular Damage in Rats

**DOI:** 10.1155/2018/8487248

**Published:** 2018-04-26

**Authors:** Rafa S. Almeer, Ahmed E. Abdel Moneim

**Affiliations:** ^1^Department of Zoology, College of Science, King Saud University, Riyadh, Saudi Arabia; ^2^Department of Zoology and Entomology, Faculty of Science, Helwan University, Cairo, Egypt

## Abstract

In the present investigation, the effect of olive leaf extract (OLE) on testicular damage induced in rats by an intraperitoneal injection of cisplatin (*cis*-diamminedichloroplatinum (CDDP)) at a dose of 5 mg/kg was tested. Rats were randomly divided into 4 groups: control, CDDP, OLE, and OLE + CDDP. After 5 days of CDDP treatment, body and testicular weights, histopathological alteration, and serum male sex hormone levels were determined. In addition to the biochemical and immunohistochemical changes in the testes, CDDP caused the disorganization of germinal epithelium and apoptosis by inducing Bax and inhibiting Bcl-2 protein expression. Testicular weights, catalase, serum testosterone, testicular enzymatic (including glutathione peroxidase, glutathione reductase, and superoxide dismutase) along with nonenzymatic (glutathione) antioxidants, and levels of luteinizing and follicle-stimulating hormones were significantly reduced in addition to a significant increase in testicular malondialdehyde and nitrite/nitrate levels when compared with the control group. OLE treatment markedly attenuated both biochemical and histopathological changes. The reproductive beneficial effects of OLE were mediated, at least partly, by inducing the nuclear factor erythroid 2-related factor 2 (Nrf2)/heme oxygenase 1 (HO-1) pathway.

## 1. Introduction

Cisplatin, *cis*-diamminedichloroplatinum (CDDP), with the molecular formula *cis*-[Pt(NH_3_)_2_Cl_2_], is widely used as a standard antineoplastic drug for treating various cancers, including bladder, lung, neck, head, and testicular cancers [1, 2]. CDDP is a DNA-alkylating molecule that exerts its antitumor activity by inducing DNA crosslinks and DNA double-strand breaks; both these actions suppress DNA transcription and replication, leading to programmed cell death/apoptosis [3]. Furthermore, CDDP induces oxidative stress by producing reactive oxygen species (ROS) that promote cellular damage and necrosis through the lipid peroxidation of tissues, DNA lesions, and protein denaturation [4]. Although CDDP is a highly effective chemotherapeutic agent, its use is mainly limited by 2 factors: resistance development to CDDP and severe toxicity to normal tissues, especially nephrotoxicity, neurotoxicity, and testicular damage [5].

The mechanism underlying CDDP-induced testicular damage includes physiological and pathohistological disturbances resulting from oxidative stress and DNA damage [4]. Hence, several antioxidants have been examined against CDDP-induced testicular damage. For example, arjunolic acid, a natural triterpenoid saponin isolated from the bark of *Terminalia arjuna* tree, significantly protected against CDDP-induced oxidative stress and inflammation in testicular tissues of rats [2]. Resveratrol administration also ameliorated CDDP-induced epididymal oxidative stress along with testicular damage, inhibited steroidogenesis and spermatogenesis, and restored normal testicular structure [3].

Olive (*Olea europaea*, Oleaceae) plant is a phytoestrogen-containing longevous tree that is socioeconomically and culturally valuable to inhabitants of the Mediterranean region. The leaves of this plant contain many flavonoid and polyphenolic compounds that possess antioxidant, anti-inflammatory, anticancer, antidiabetic, gastroprotective, and wound healing properties [6, 7]. Recently, Al-Quraishy et al. [7] reported that oleuropein is the most abundant molecule, representing 86.9% of total identified compounds in olive leaf extract (OLE) and exerts good antioxidant and anti-inflammatory activities. In this study, we examined the potential impact of OLE on CDDP-induced testicular impairment in rats. Additionally, we explored the involvement of the nuclear factor erythroid 2-related factor 2 (Nrf2)/heme oxygenase 1 (HO-1) pathway in the protective effects of OLE.

## 2. Materials and Methods

### 2.1. Preparation of OLE

Dried olive leaves were collected from a local market (El-Yamani Corner, Riyadh, Kingdom of Saudi Arabia). The leaves were identified and confirmed by a specialized taxonomist (Department of Botany, College of Science, King Saud University, Saudi Arabia). They were cleaned and homogenized into a fine powder (50 g), which was then extracted with 500 mL of 70% methanol at 4°C with stirring every 4 h for 48 h. After filtration, the methanol was evaporated to semidryness in a vacuum evaporator, and the extract was lyophilized. The obtained OLE was dissolved in distilled water at a final concentration of 300 mg/mL and stored at −20°C in the dark until use in this study.

### 2.2. Experimental Protocol

Adult healthy Wistar male rats were individually housed in polypropylene cages, acclimated for 5 days before initiating the experiments, in a temperature-controlled room (22 ± 2°C) under the normal light/dark cycle of the day with unrestricted access to water and standard rodent diet. All experiments were conducted according to the guidelines of the National Program for Science and Technology of Faculty of Science, King Saud University. The study protocol was approved (IRB number: K.S.U-2017-750/PI) by the Ethical Committee of King Saud University (Riyadh, Kingdom of Saudi Arabia).

The rats (*n* = 7/group) were randomized to receive saline, CDDP, OLE, or OLE + CDDP. Cisplatin was acquired from Sigma-Aldrich (St. Louis, MO, USA). CDDP was intraperitoneally administered at 5 mg/kg on day 1, and OLE was orally administered at 300 mg/kg daily for 5 consecutive days. This dose of CDDP was selected based on published reports [1, 5]. The dosing regimen for OLE was selected based on a report by Al-Quraishy et al. [7] showing that this is a safe dosing regimen.

The rats were then sacrificed by the intravenous administration of sodium pentobarbital (300 mg, Sigma-Aldrich) 24 h after the last OLE administration. Blood was collected, and the testes were removed and washed in ice-cold 0.01 M phosphate buffer (pH 7.4). After drying well with a filter paper, the left testis was weighed and homogenized to give a 10% (*w*/*v*) homogenate. Protein concentration of the samples was determined according to the method of Lowry et al. [8].

### 2.3. Changes in the Testis Index of Rats

The relative weight of the testis was calculated according to the weight of the left testis as follows: (weight of left testis/body weight) × 100.

### 2.4. Estimation of Serum Sex Hormones

After the collection of blood samples, the samples were centrifuged (5000 rpm for 10 min at 4°C) to separate the serum, and fresh serum was used immediately for the analysis of sex hormones. Serum testosterone and luteinizing hormone (LH) and follicle-stimulating hormone (FSH) of the rats were quantitatively measured by ELISA using specific kits (MyBioSource, CA, USA). The experiment was performed as per the manufacturer's instructions.

### 2.5. Oxidative Stress Markers

Malondialdehyde (MDA), the main end product formed due to lipid peroxidation in the tissues, serves as a marker for lipid peroxidation. To determine MDA level in the testes, the homogenate was reacted with thiobarbituric acid using the method of Ohkawa et al. [9]. Nitrite level in the homogenate was measured by the method of Green et al. [10]. Testicular reduced glutathione (GSH) was determined using Ellman's reagent as described previously [11].

### 2.6. Antioxidant Status

Testicular antioxidant enzyme activities were determined as markers for the assessment of oxidant/antioxidant balance in the testis. Superoxide dismutase (SOD) activity was measured by the nitroblue tetrazolium reduction assay [12]. Catalase (CAT) activity was also assayed by reacting the testis homogenate with H_2_O_2_. The consumption of H_2_O_2_ was quantified spectrophotometrically at 340 nm for 120 s at 30 s intervals [13]. Both SOD and CAT activities are presented as units/mg protein. Glutathione reductase (GSH-R) was determined by the method of Dringen and Gutterer [14], where 1 mL of a mixture containing 0.05 M phosphate buffer (pH 7.0), 1 mM EDTA, 10 mM oxidized glutathione (GSSG), and 0.1 mM NADPH was used. GSH-R activity was determined by change in NADPH concentration with time after adding the testis homogenate. Finally, glutathione peroxidase (GSH-Px) activity was assayed as described by Paglia and Valentine [15]. In this method, GSSG produced from GSH due to GSH-Px in the presence of NADPH and GSH-R was measured. GSH-Px activity was computed from the change in NADPH concentration with time using *ɛ* = 6270 M^−1^·cm^−1^.

### 2.7. Inflammation Markers

The extent of inflammation in testis samples was estimated by measuring IL-1*β* and TNF-*α* levels using commercial kits according to the manufacturer's instructions (Merck Millipore, Australia).

### 2.8. Quantitative Real-Time PCR

RNA in the testis samples was isolated utilizing the TRIzol reagent (Invitrogen, CA, USA), and 1 *μ*g of the isolated RNA was used as a template together with random primers to synthesize cDNA utilizing Thermo Scientific Maxima First Strand cDNA Synthesis Kit for RT-qPCR. Each cDNA sample was run in triplicate for real-time PCR analysis. *GAPDH* (accession number: NM_017008.4; sense: 5′-GCATCTTCTTGTGCAGTGCC-3′; antisense: 5′-GATGGTGATGGGTTTCCCGT-3′) served as a housekeeping gene. Real-time PCR reactions were performed utilizing the Power SYBR Green Applied Biosystems 7500 System (Life Technologies, CA, USA) at 94°C for 4 min, followed by 42 cycles at 94°C for 1 min, at 60°C for 1 min, and then held for the final phase at 72°C for 10 min. Gene expression analysis employed the 2^−ΔΔCt^ method according to Pfaffl [16]. The PCR primers for the following genes were synthesized by Invitrogen: *SOD2* (superoxide dismutase 2, mitochondrial; accession number: NM_001270850.1; sense: 5′-AGCTGCACCACAGCAAGCAC-3′; antisense: 5′-TCCACCACCCTTAGGGCTCA-3′), *CAT* (accession number: NM_012520.2; sense: 5′-TCCGGGATCTTTTTAACGCCATTG-3′; antisense: 5′-TCGAGCACGGTAGGGACAGTTCAC-3′), *GPx1* (accession number: NM_030826.4; sense: 5′-CAGTCCACCGTGTATGCCTT-3′; antisense: 5′-GTAAAGAGCGGGTGAGCCTT-3′), *Nrf2* (accession number: NM_031789.2; sense: 5′-GGTTGCCCACATTCCCAAAC-3′; antisense: 5′-GGCTGGGAATATCCAGGGC-3′), *HO-1* (accession number: NM_012580.2; sense: 5′-GCGAAACAAGCAGAACCCA-3′; antisense: 5′-GCTCAGGATGAGTACCTCCC-3′), *Bcl-2* (accession number: NM_016993.1; sense: 5′-CTGGTGGACAACATCGCTCTG-3′; antisense: 5′-GGTCTGCTGACCTCACTTGTG-3′), and *Bax* (accession number: NM_017059.2; sense: 5′-GGCGAATTGGCGATGAACTG-3′; antisense: 5′-ATGGTTCTGATCAGCTCGGG-3′).

### 2.9. Histological Examination

Testis tissues were immersed in neutral buffered formalin (4%), dehydrated in 70% ethanol, and embedded in paraffin. The tissue blocks were sectioned at 4-5 *μ*m and stained with hematoxylin-eosin. The sections were visualized under a Nikon optical microscope (Eclipse E200-LED, Tokyo, Japan). Only seminiferous tubules with their epithelium cycle between stages 9 and 13 characterized by a single generation of spermatids and 2 generations of primary spermatocytes were observed.

### 2.10. Immunohistochemistry

The paraffin-embedded sections were mounted on charged slides, deparaffinized, and washed with phosphate-buffered saline. The antigen sites were unmasked by heating, and the endogenous peroxidase was inactivated by 3% H_2_O_2_. The sections were blocked with 10% (*w*/*v*) normal goat serum for 1 h and then incubated with polyclonal rabbit anti-proliferating cell nuclear antigen (PCNA), Bcl-2, and Bax antibodies (Santa Cruz Biotechnology, Santa Cruz, CA, USA) overnight at 4°C. Afterward, all samples were incubated with biotinylated secondary antibodies (1 : 1000) for 30 min at 37°C. The specific protein immunoreactivity was visualized by the chromogen 3,3′-diaminobenzidine tetrachloride method under 400x magnification (Nikon Eclipse E200-LED, Tokyo, Japan) with an Olympus camera.

### 2.11. Statistical Analysis

Results are expressed as the mean ± standard deviation. Statistical analyses were performed using one-way ANOVA with the SPSS (version 20.0) followed by Tukey's post hoc test. A *p* value of less than 0.05 was considered as a criterion for a statistically significant difference.

## 3. Results

### 3.1. Body and Testis Weights

**Figure 1:**
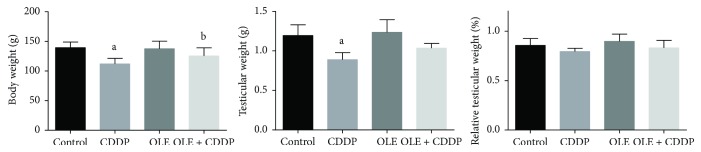
Potential effects of olive leaf extract (OLE) treatment on body weight, testis weight, and relative testicular weight in rats treated with cisplatin (CDDP). All data are expressed as the mean ± SEM (*n* = 7). ^a^Significant change from the control group at *p* < 0.05; ^b^significant change from the CDDP group at *p* < 0.05 using Tukey's post hoc test.

Upon CDDP injection, no death was observed in the rats. However, the body and testicular weights were significantly (*p* < 0.05) lower than the control weights (Figure 1). However, relative testis weight that was obtained at the end of the experiment was lower (nonsignificant) than control. The rats treated with OLE + CDDP showed significantly (*p* < 0.05) higher body weights than CDDP-treated rats.

### 3.2. Serum Concentrations of Sex Hormones

**Figure 2:**
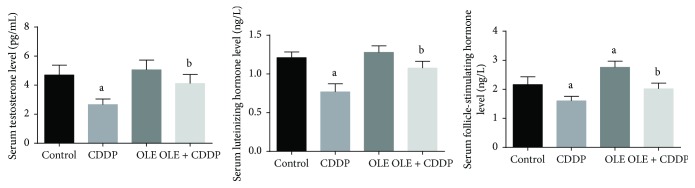
Effects of olive leaf extract (OLE) treatment on testosterone, luteinizing hormone, and follicle-stimulating hormone levels in the serum of rats treated with cisplatin (CDDP). All data are expressed as the mean ± SEM (*n* = 7). ^a^Significant change from the control group at *p* < 0.05; ^b^significant change from the CDDP group at *p* < 0.05 using Tukey's post hoc test.

Serum testosterone, LH, and FSH concentrations were significantly (*p* < 0.05) lower in the CDDP-treated rats than in the control rats (Figure 2). Serum sex hormone concentrations in the OLE + CDDP treatment group increased significantly (*p* < 0.05) by the end of the experiment and returned to normal values.

### 3.3. Oxidative Stress Parameters

**Figure 3:**
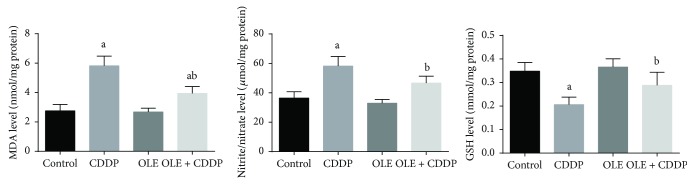
Effects of olive leaf extract (OLE) treatment on malondialdehyde (MDA), nitrite/nitrate, and glutathione (GSH) content in the testis of rats treated with cisplatin (CDDP). All data are expressed as the mean ± SEM (*n* = 7). ^a^Significant change from the control group at *p* < 0.05; ^b^significant change from the CDDP group at *p* < 0.05 using Tukey's post hoc test.

There was a significant (*p* < 0.05) elevation in testicular MDA and nitrite/nitrate levels with concomitant depletion in GSH concentration in the CDDP group compared with the control group, indicating the oxidative action of CDDP on testicular tissues. Conversely, OLE treatment with CDDP prevented changes in MDA, nitrite/nitrate, and GSH levels, demonstrating the antioxidant activity of OLE (Figure 3).

**Figure 4:**
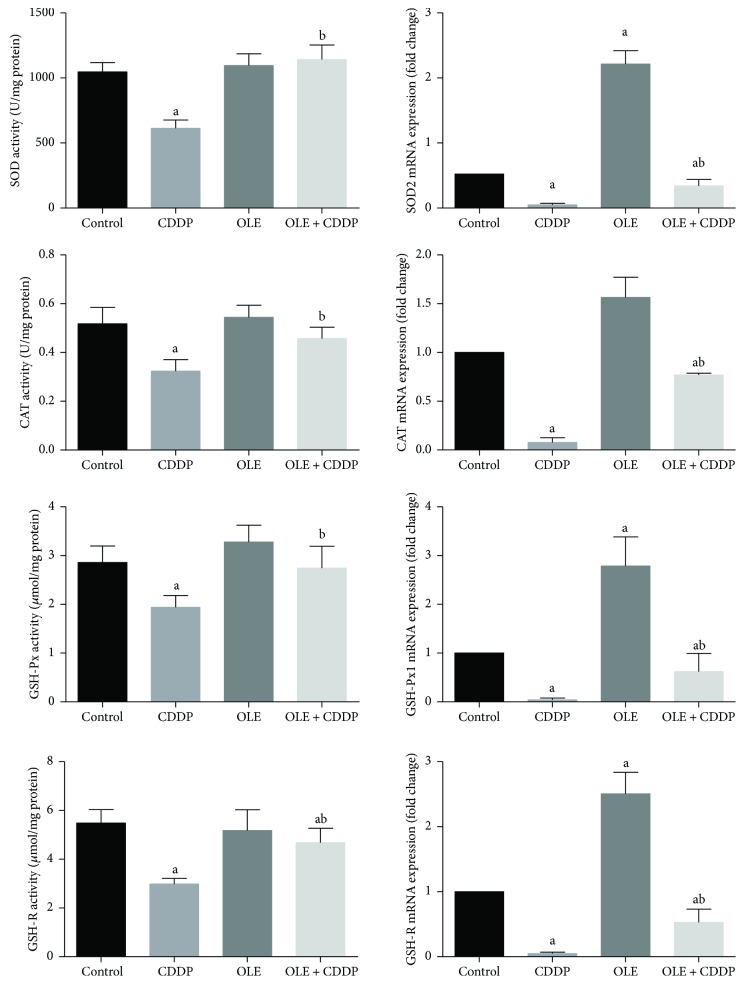
Effects of olive leaf extract (OLE) treatment on superoxide dismutase (SOD), catalase (CAT), glutathione peroxidase (GSH-Px), and glutathione reductase (GSH-R) activities and their corresponding mRNA expression in the testis of rats treated with cisplatin (CDDP). Data of antioxidant enzyme activities are expressed as the mean ± SEM (*n* = 7), whereas mRNA expression data are expressed as the mean ± SEM of triplicate assays, normalized to the *GAPDH* mRNA level, and shown as fold change (in log2 scale) relative to the control mRNA levels. ^a^Significant change from the control group at *p* < 0.05; ^b^significant change from the CDDP group at *p* < 0.05 using Tukey's post hoc test.

Activities of SOD, CAT, GSH-Px, and GSH-R were significantly (*p* < 0.05) lower in the CDDP-treated rats than in the control rats (Figure 4). However, these activities were only partially attenuated in rats in the OLE + CDDP group compared with those in the control group but significantly (*p* < 0.05) higher than those in CDDP-injected rats. The gene expression of antioxidant enzymes (SOD, CAT, GSH-Px, and GSH-R) was lower in the testis tissues of rats in the CDDP group than in the control group. Interestingly, OLE treatment significantly (*p* < 0.05) increased the expression of all enzymes, except CAT, compared to control. OLE treatment significantly mitigated CDDP-induced oxidative stress in the testes (Figure 4).

### 3.4. Nrf2 and HO-1 Overexpression Protects against CDDP-Induced Testicular Oxidative Stress

**Figure 5:**
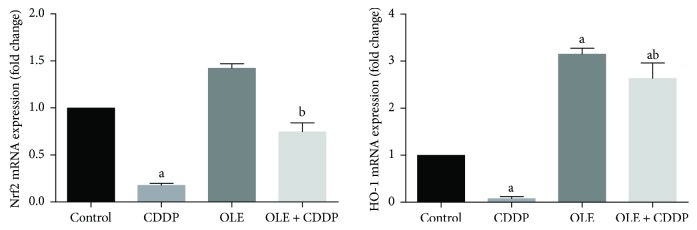
Effects of olive leaf extract (OLE) treatment on nuclear factor erythroid 2-related factor (Nrf2) and heme oxygenase-1 (HO-1) mRNA expression in the testis of rats treated with cisplatin (CDDP). Data of the mRNA expression are expressed as the mean ± SEM of triplicate assays, normalized to the *GAPDH* mRNA level, and shown as fold change (in log2 scale) relative to the control mRNA levels. ^a^Significant change from the control group at *p* < 0.05; ^b^significant change from the CDDP group at *p* < 0.05 using Tukey's post hoc test.

mRNA expression levels of Nrf2 and HO-1 were significantly (*p* < 0.05) lower in the CDDP-treated group than in the control group (Figure 5). HO-1 mRNA expression was significantly higher in the OLE group than in the control group. Both Nrf2 and HO-1 mRNA levels were higher in the OLE + CDDP group than in the CDDP; moreover, HO-1 mRNA level was (*p* < 0.05) upregulated compared to the control group.

### 3.5. Inflammation Is Involved in CDDP-Induced Testicular Damage

**Figure 6:**
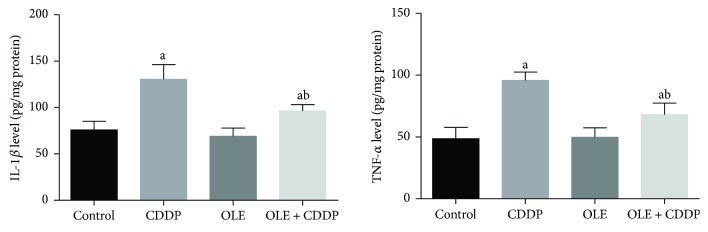
Effects of olive leaf extract (OLE) treatment on TNF-*α* and IL-1*β* levels in the testis of rats treated with cisplatin (CDDP). All data are expressed as the mean ± SEM (*n* = 7). ^a^Significant change from the control group at *p* < 0.05; ^b^significant change from the CDDP group at *p* < 0.05 using Tukey's post hoc test.

To elucidate whether OLE is able to reduce CDDP-induced testicular inflammation, we determined inflammatory markers in the testis homogenates. As illustrated in Figure 6, CDDP administration significantly (*p* < 0.05) elevated IL-1*β* and TNF-*α* levels in the testis compared to control. However, treatment with OLE significantly (*p* < 0.05) inhibited the production of these inflammatory markers.

### 3.6. Histopathological Findings

**Figure 7:**
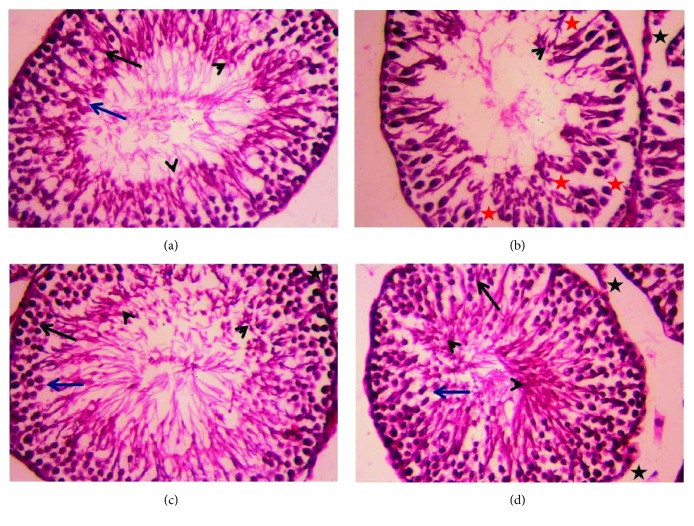
Light micrographs of testicular tissues of rats treated with olive leaf extract (OLE) and cisplatin (CDDP). (a) Photomicrograph of the testicular tissue of the control group showing healthy seminiferous tubules at all stages of spermatogenic cells (primary spermatocyte “black arrow” and spermatids “blue arrow”) and the interstitial cells with Leydig cells (black star) filling the space between the seminiferous tubules. (b) Photomicrograph of the testicular tissue of rats treated with CDDP showing degenerative alterations (red star) in spermatogenic cells and the detachment of the spermatogenic epithelium. (c) Photomicrograph of the testicular tissue of rats treated with OLE alone showing a healthy histological structure. (d) Photomicrograph of the testicular tissue of rats treated with OLE and CDDP showing a recovery of spermatogenic epithelium in most seminiferous tubules. Sections were stained with hematoxylin and eosin (400x).

Although CDDP injection caused testicular atrophy with a severe degeneration of the germinal epithelium in seminiferous tubules with many residual bodies and the disorganization and shedding of the germinal epithelium into the lumina (Figure 7(b)), OLE treatment ameliorated these changes in the seminiferous epithelium (Figure 7(d)). Control and OLE-treated rats appeared to have normal testicular structures with an orderly pattern of germinal epithelial and Sertoli cells (Figures 7(a) and 7(c)).

### 3.7. Bcl-2 Overexpression Protects against CDDP-Induced Testicular Apoptosis

**Figure 8:**
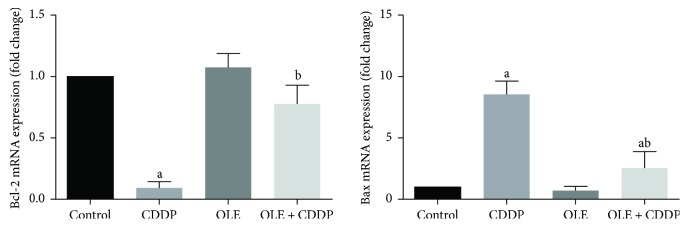
Effects of olive leaf extract (OLE) treatment on Bcl-2 and Bax mRNA expression in the testis of rats treated with cisplatin (CDDP). Data of the mRNA expression are expressed as the mean ± SEM of triplicate assays, normalized to the *GAPDH* mRNA level, and shown as the fold change (in log2 scale) relative to the control mRNA levels. ^a^Significant change from the control group at *p* < 0.05; ^b^significant change from the CDDP group at *p* < 0.05 using Tukey's post hoc test.

In the present study, we also investigated whether the testicular protective effects of OLE are associated with its antiapoptotic activity; Bcl-2 and Bax mRNA expression levels in the testis were examined. Bcl-2 mRNA expression was significantly (*p* < 0.05) downregulated (Figure 8), whereas Bax mRNA expression was significantly (*p* < 0.05) upregulated in CDDP-treated rats. However, OLE treatment significantly downregulated Bax and upregulated Bcl-2 mRNA expression compared to CDDP treatment.

**Figure 9:**
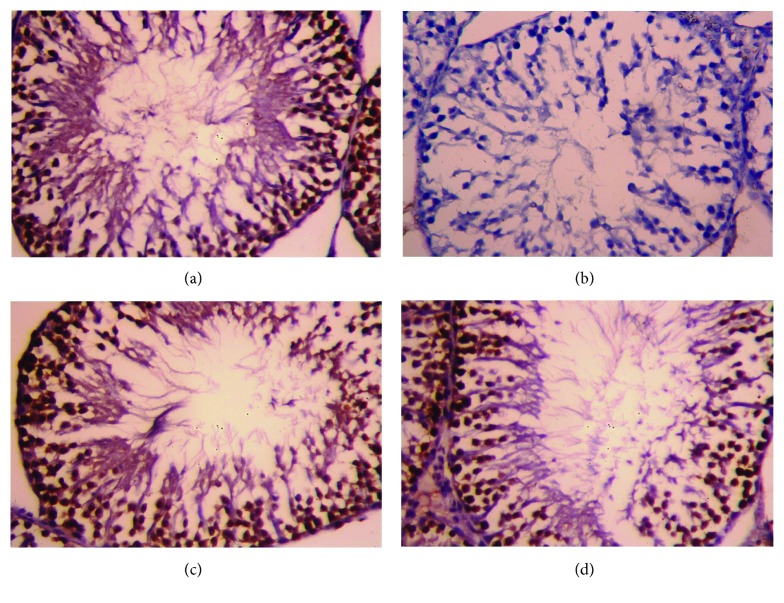
Testicular expression of Bcl-2 protein was detected using immunohistochemical staining in (a) control, (b) cisplatin (CDDP), (c) olive leaf extract (OLE), and (d) OLE + CDDP groups. In the control and OLE groups, Bcl-2-positive brown-stained cells were moderately to strongly immunostained. However, many testicular cells were weakly stained with brown color due to CDDP. In the OLE + CDDP group, the number of Bcl-2-positive cells was markedly increased. (400x).

**Figure 10:**
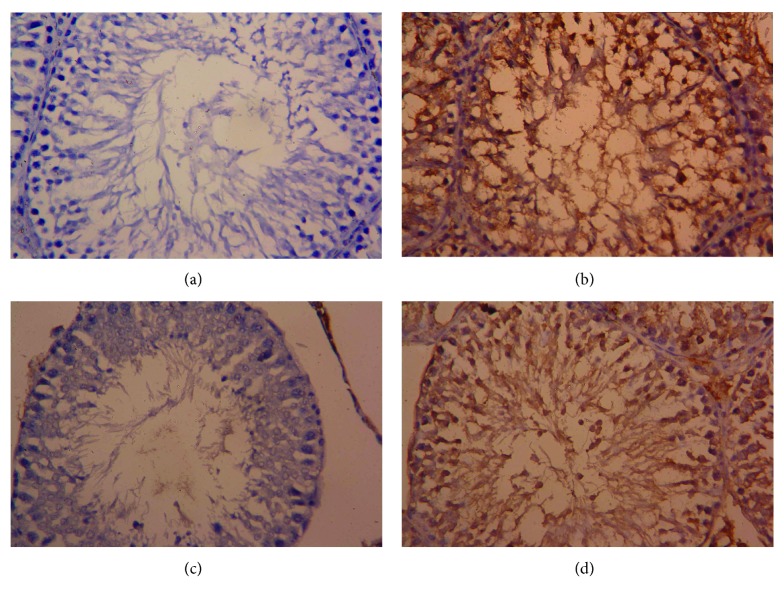
Testicular expression of Bax protein was detected using immunohistochemical staining in (a) control, (b) cisplatin (CDDP), (c) olive leaf extract (OLE), and (d) OLE + CDDP groups. In the control and OLE groups, Bax-positive brown-stained cells were sparse and weakly immunostained. However, many testicular cells exhibited apoptosis and were stained brown (Bax positive) due to CDDP. In the OLE + CDDP group, the number of Bax-positive cells was markedly increased. (400x).

Consistent with RT-PCR results, immunohistochemistry showed that the number of Bcl-2-positive cells in the testicular tissues of CDDP-treated rats was markedly decreased (Figure 9(b)), whereas moderate to strong immunoreaction was observed for Bax (Figure 10(b)). The administration of OLE to rats injected with CDDP exhibited an increase in Bcl-2-positive spermatogenic cells (Figure 9(d)) with moderate immunoreaction for the proapoptotic protein, Bax (Figure 10(d)).

### 3.8. CDDP-Induced Downregulation in PCNA Expression

**Figure 11:**
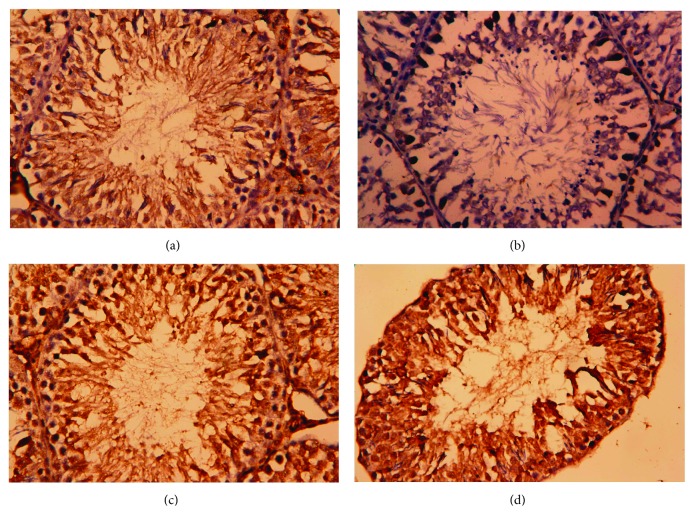
Testicular expression of proliferating cell nuclear antigen (PCNA) protein was detected using immunohistochemical staining in (a) control, (b) cisplatin (CDDP), (c) olive leaf extract (OLE), and (d) OLE + CDDP groups. In the control and OLE groups, PCNA-positive brown-stained cells were moderately to strongly immunostained. However, many testicular cells were weakly stained with brown color due to CDDP. In the OLE + CDDP group, the number of PCNA-positive cells was markedly increased. (400x).

Immunohistochemical analysis revealed a lower expression of PCNA protein in the CDDP group than in the control group (Figure 11(b)). However, OLE treatment significantly increased the number of PCNA-positive cells (Figure 11(d)).

## 4. Discussion

Although CDDP is the most used antitumor agent in the chemotherapy of various cancers, its use is limited due to its adverse effects on the kidney, nervous system, and testis. Testicular dysfunction is the most reported consequence of CDDP toxicity because of the high proliferation rate of testicular cells. CDDP has been reported to cause sperm impairment, spermatogenic apoptosis, and abnormality in Leydig cells in experimental animals [17, 18]. CDDP administration to rats significantly decreases testis weight and disrupts male sexual hormone levels [4]. Furthermore, upon microscopic examination, notable degeneration, necrosis/apoptosis, and reduction in the circumference of seminiferous tubules and spermatogenic cell thickness have been reported after CDDP treatment. Testis weight depends on the mass of differentiated spermatogenic cells, and its structural and functional integrity requires the adequate biosynthesis of male sex hormones. Thus, a decline in testis weight in CDDP-treated rats reveals reduced spermatogenesis and steroidogenesis [17]. In the current study, CDDP administration significantly reduced male reproductive hormone levels. This may be attributed to reduced Leydig cells that produce gonadotropin, along with depressed mitochondrial side-chain cleavage and cytochrome P-450 activity [19]. CDDP also affects the function of Sertoli cells and decreases the expression of androgen-binding proteins [20]. Indeed, hormonal perturbation caused by cisplatin is mediated by its effects on the hypothalamic-pituitary-gonadal axis [21]. According to the obtained results, OLE treatment significantly increased testis weight and levels of male sex hormones (testosterone, LH, and FSH) compared to CDDP administration. Recent studies demonstrated that herbal extracts prevent CDDP-induced reproductive injury because of their antioxidant constituents [17, 22].

In the current study, CDDP-induced gonadal toxicity and tissue atrophy were due to increased ROS production and depleted enzymatic and nonenzymatic testicular antioxidant defense molecules. CDDP is known to disturb the oxidant/antioxidant balance in the testicular tissue [23]. In the current study, CDDP significantly elevated MDA and nitrite/nitrate levels and depleted GSH content and activities of SOD, CAT, GSH-R, and GSH-Px in the testis, indicating that the enzymatic and nonenzymatic antioxidant molecules were inadequate for scavenging free radicals produced due to CDDP. MDA serves as a marker for oxidative stress due to the peroxidation of cellular polyunsaturated fatty acids. Both nitrate and nitrite levels have been used as indexes of nitric oxide generation and nitrosative stress [24]. GSH is the most abundant cellular sulphydryl molecule that interacts with oxidizing compounds, and a reduction in its cellular content has been considered as an indication of oxidative stress [25]. SOD presents the first preventive antioxidant enzyme that neutralizes singlet oxygen (^1^O_2_) and spontaneously dismutates superoxide radicals (O^−^_2_) to H_2_O_2_. The decomposition of H_2_O_2_ is successfully accomplished by CAT, thereby preventing lipid peroxidation. GSH-Px together with GSH catalyzes the reduction of H_2_O_2_ and lipid peroxides, whereas GSH-R promotes the NADPH-driven conversion of GSSG to GSH [26]. A depletion of these antioxidant enzymes and molecules could be associated with an overwhelming accumulation of H_2_O_2_ that suppresses testicular antioxidant defense systems. However, OLE treatment attenuated testicular oxidative stress and restored the antioxidant defense system in the testicular tissue, indicating that OLE prevents CDDP-induced oxidative stress and reproductive damage. The antioxidant activity of OLE has been well documented previously, and Bouaziz et al. attributed this effect to the phenolic constituents, oleuropein, luteolin, hydroxytyrosol, and orthodiphenols [27]. Servili et al. [28] mentioned that olive phenols modulate the cellular redox status by enzymes.

Nrf2 is a basic leucine zipper transcription factor that protects the cell against oxidative stress through the antioxidant response element-mediated induction of various phase 2 metabolism and antioxidant enzymes, including efflux transporters, heat shock proteins, and proteasomal degradation enzymes [29]. HO-1 is a stress-responsive enzyme that transforms heme into biliverdin and free iron along with carbon monoxide [30]. In cases of elevated oxidative stress due to exposure to a diverse array of toxic insults, HO-1 is induced as a beneficial response in cells. Moreover, the promotion of HO-1 drastically decreases CDDP-induced cytotoxicity by regulating autophagy [24]. The current investigation suggests that the beneficial effect of OLE is due to an induction of Nrf2 and HO-1, thereby maintaining the transcriptional activation status of detoxification enzymes and drug transporters and suppressing inflammation; these effects enhance the survival of germinal epithelial cells despite CDDP administration.

Inflammation is involved in CDDP-induced tissue toxicity [31]. CDDP triggers the NF-*κ*B pathway, thereby promoting the expression of a series of inflammatory cytokines, including TNF-*α* and IL-1*β* [32]. OLE treatment prevented inflammatory cytokine production in CDDP-induced reproductive toxicity. The findings of the current investigation are consistent with a previous study by Al-Quraishy et al. [7], in which OLE prevented gastric ulcer by reducing the production of TNF-*α*, IL-1*β*, and other proinflammatory cytokines. OLE treatment during chemotherapy can prevent TNF-*α* and IL-1*β* overexpression [33].

Germinal epithelium apoptosis has been documented as a possible mechanism for the testicular damage following CDDP treatment. In the current study, CDDP injection upregulated Bax and downregulated Bcl-2 expression in the testis. Bcl-2 is located in the outer membrane of the mitochondria, which promotes cell survival and counters the actions of the proapoptotic protein, Bax, thereby maintaining mitochondrial membrane integrity. However, Bax promotes mitochondrial permeabilization causing a discharge of both cytochrome C and ROS from the mitochondria into the cytoplasm under conditions of oxidative stress. In the cytoplasm, cytochrome C interacts with the apoptotic protease-activating factor 1 and forms an apoptosome, which finally activates caspase-3, the key regulator in the execution of apoptosis. This leads to DNA fragmentation, chromatin condensation, and biomembrane protein destruction [29, 34]. In the current study, an alternation in the mRNA levels of Bax and Bcl-2 was observed in rats injected with CDDP. The downregulation of Bcl-2 associates with a loss of survival signals, but the upregulation of Bax can be a marker of apoptosis via the intrinsic pathway. Our results were similar to the observation obtained by Cao et al. [35], which they found that CDDP-induced apoptosis in human nasopharyngeal carcinoma CNE-2 cells via upregulating Bax and downregulating Bcl-2. In the present study, OLE prevented apoptosis by upregulating Bcl-2 and downregulating Bax in the testicular tissue of CDDP-treated rats. Similarly, Al-Quraishy et al. [7] reported that the antiapoptotic activity of OLE is due to its antioxidant and anti-inflammatory properties.

## 5. Conclusions

In conclusion, CDDP injection in rats induces histopathological alterations and apoptosis in testicular tissues through oxidative stress induction as evidenced by elevated lipid peroxidation and nitrite/nitrate generation and depleted enzymatic and nonenzymatic antioxidants. However, OLE treatment protected against CDDP-induced testicular toxicity owing to its antioxidant, anti-inflammatory, and antiapoptotic properties. The present study also revealed that increased Nrf2 and HO-1 expression could be an effective strategy for preventing CDDP-induced testicular injury.
